# A rare combination of tumor-induced osteomalacia caused by sinonasal glomangiopericytoma and coexisting parathyroid adenoma: case report and literature review

**DOI:** 10.1186/s12902-022-00934-7

**Published:** 2022-01-28

**Authors:** Agnieszka Brociek-Piłczyńska, Dorota Brodowska-Kania, Kornel Szczygielski, Małgorzata Lorent, Grzegorz Zieliński, Piotr Kowalewski, Dariusz Jurkiewicz

**Affiliations:** 1 Department of Otolaryngology with Division of Cranio-Maxillo-Facial Surgery, Military Institute of Medicine, Szaserów 128, 04-141, Warsaw, Poland; 2 Department of Endocrinology and Isotope Therapy, Military Institute of Medicine, Warsaw, Poland; 3 Department of Pathology, Military Institute of Medicine, Warsaw, Poland; 4 Department of Neurosurgery, Military Institute of Medicine, Warsaw, Poland; 5 Department of General Surgery, Military Institute of Aviation Medicine, Warsaw, Poland

**Keywords:** Tumor-induced osteomalacia, Oncogenic osteomalacia, Glomangiopericytoma, Sinonasal-type hemangiopericytoma, FGF-23, Hyperparathyroidism, Parathyroid adenoma, Case report

## Abstract

**Background:**

Tumor-induced osteomalacia (TIO) is a rare, acquired disease of renal phosphate wasting and disturbed vitamin D homeostasis as a result of the action of a phosphaturic protein – FGF-23, produced by a neoplasm. Although the clinical and biochemical profile of the syndrome is characteristic, it remains underreported and unrecognized by clinicians. Hyperparathyroidism is rarely associated with oncogenic osteomalacia, but it should be considered because of potentially life-threatening hypophosphatemia caused by both conditions.

**Case presentation:**

We report a case of a 42-year-old woman admitted to the Department of Otolaryngology of the Military Institute of Medicine in Warsaw for the endoscopic resection of hormonally active glomangiopericytoma extending into the anterior skull base. She presented with a 5-year history of musculoskeletal pain and progressive weakness of the extremities which finally led her to become bedridden. After the excision of the tumor her symptoms and laboratory results gradually improved except increasing PTH serum levels. Further examination revealed a parathyroid proliferative tumor, which was surgically removed. The patient walked without aids at follow-up 16 months after the surgery.

**Conclusions:**

This case is unusual because of tumor-induced osteomalacia and parathyroid adenoma occurring concomitantly. Further investigations of FGF-23 and PTH interplay should be conducted to elucidate the pathogenesis of hyperparathyroidism and tumorigenesis in some cases of TIO. By presenting this case, we wanted to remind clinicians of a rare and misdiagnosed paraneoplastic syndrome and highlight the importance of monitoring PTH concentrations during the follow-up of patients with TIO.

## Background

Tumor-induced osteomalacia (TIO), also known as oncogenic osteomalacia (OOM), is a rare paraneoplastic syndrome resulting from impaired phosphate and vitamin D metabolism and caused by tumors (most commonly of mesenchymal origin) secreting fibroblast growth factor 23 (FGF-23). In adults, the condition is manifested by bone pain, muscle weakness and fractures, which may lead to severe mobility deterioration and disability [1]. Laboratory findings are characteristic and include: hypophosphatemia, low or inappropriately normal concentrations of 1,25 dihydroxyvitamin D (1,25[OH]_2_D_3_), increased serum alkaline phosphate (ALP) activity and, typically, a normal measure of parathyroid hormone (PTH) [1–6]. Concomitant hyperparathyroidism is an uncommon feature of TIO, however, such cases are reported in the literature [1, 7, 8]. The rarity of the syndrome (fewer than 1000 cases reported in the literature [9]), non-specific clinical picture and difficulty in locating the tumor delay the diagnosis to approximately 2.5 years [10]. After the removal of co-existing tumor, a significant and rapid improvement of patient’s symptoms and biochemical abnormalities is observed [2–4, 6, 8]. We report a case of a woman with a metabolically active sinonasal glomangiopericytoma causing advanced TIO, with concurrent hyperparathyroidism in the course of parathyroid adenoma, and her clinical and laboratory response to surgical treatment.

## Case report

**Fig. 1:**
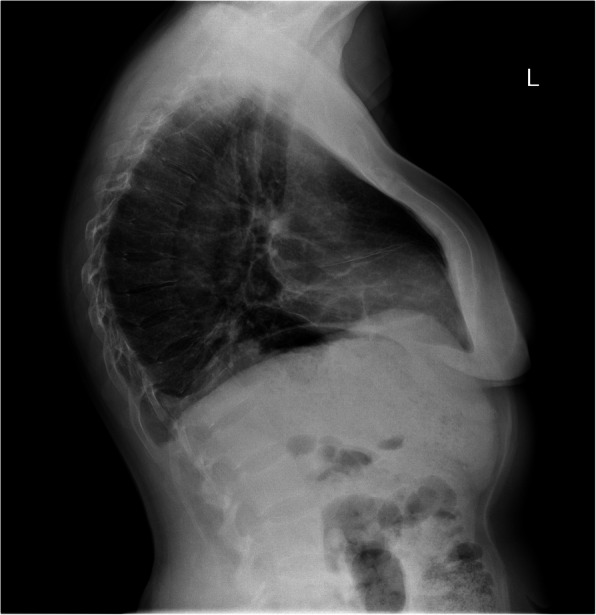
Chest radiograph showing marked kyphosis

A 42-year-old woman was admitted to the Department of Otolaryngology of the Military Institute of Medicine in Warsaw for the endoscopic resection of intranasal tumor extending into the anterior skull base. She presented with diffuse musculoskeletal pain, marked thoracic kyphosis (Fig. 1), numbness of the extremities and progressive disability leading her to be confined to bed over the last few weeks before the hospitalization. The first symptoms, i.e. osteoarticular pain and fatigue, had been noted by the patient 5 years earlier – initially they had included the shoulder and wrist joints, then gradually developed in the lumbar spine and hips causing gait abnormalities and the necessity of using crutches to walk. She reported the symptoms to be fluctuating with an improvement experienced during summer months. The only local manifestation of the tumor was infrequent left-sided nasal bleeding with the onset dated back to about 6-7 years before. She denied having nasal obstruction, rhinorrhea or headaches. She was a smoker and had no chronic diseases or relevant family history of illness.

**Fig. 2:**
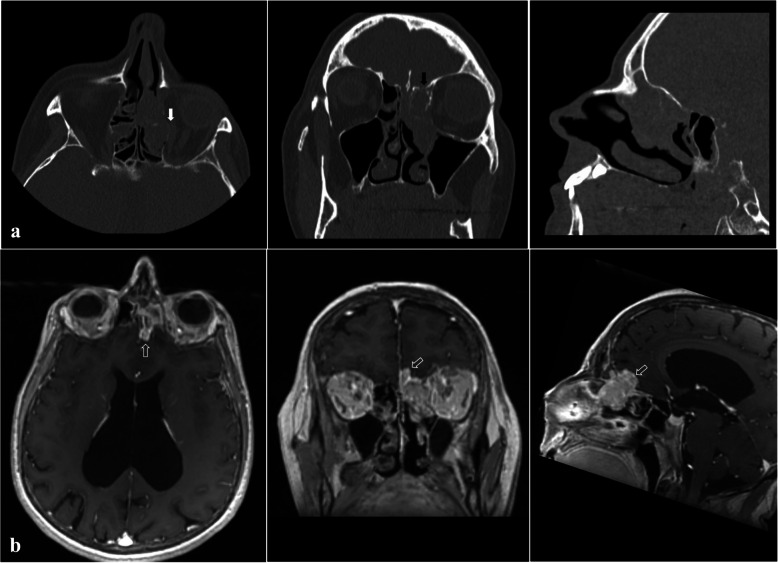
Soft tissue mass involving the fronto-ethmoid area, abutting the left rectal medius muscle (white arrow) with possible intracranial tumor spread (black arrow) visible on a CT scan (**a**). MRI confirmed the intracranial invasion of the tumor (white empty arrow) (**b**)

**Fig. 3:**
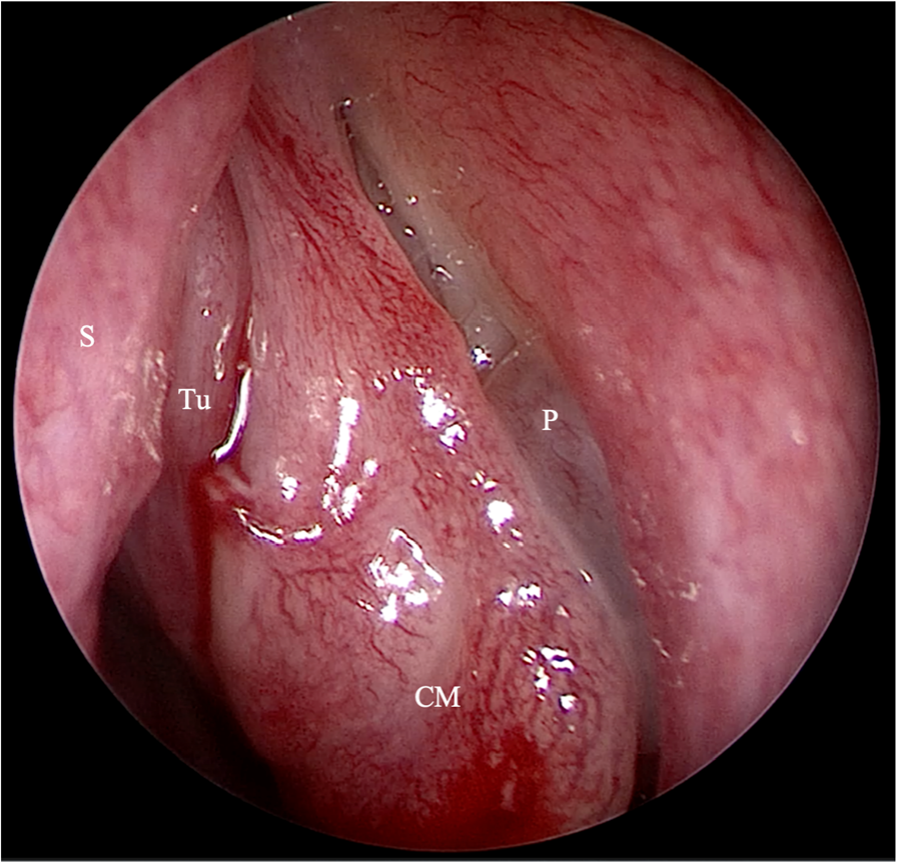
View of the operation field before an endoscopically-controlled resection. Nasal septum (S), concha media (CM), polyp in the region of the maxillary sinus ostium (P), tumor (Tu)

**Fig. 4:**
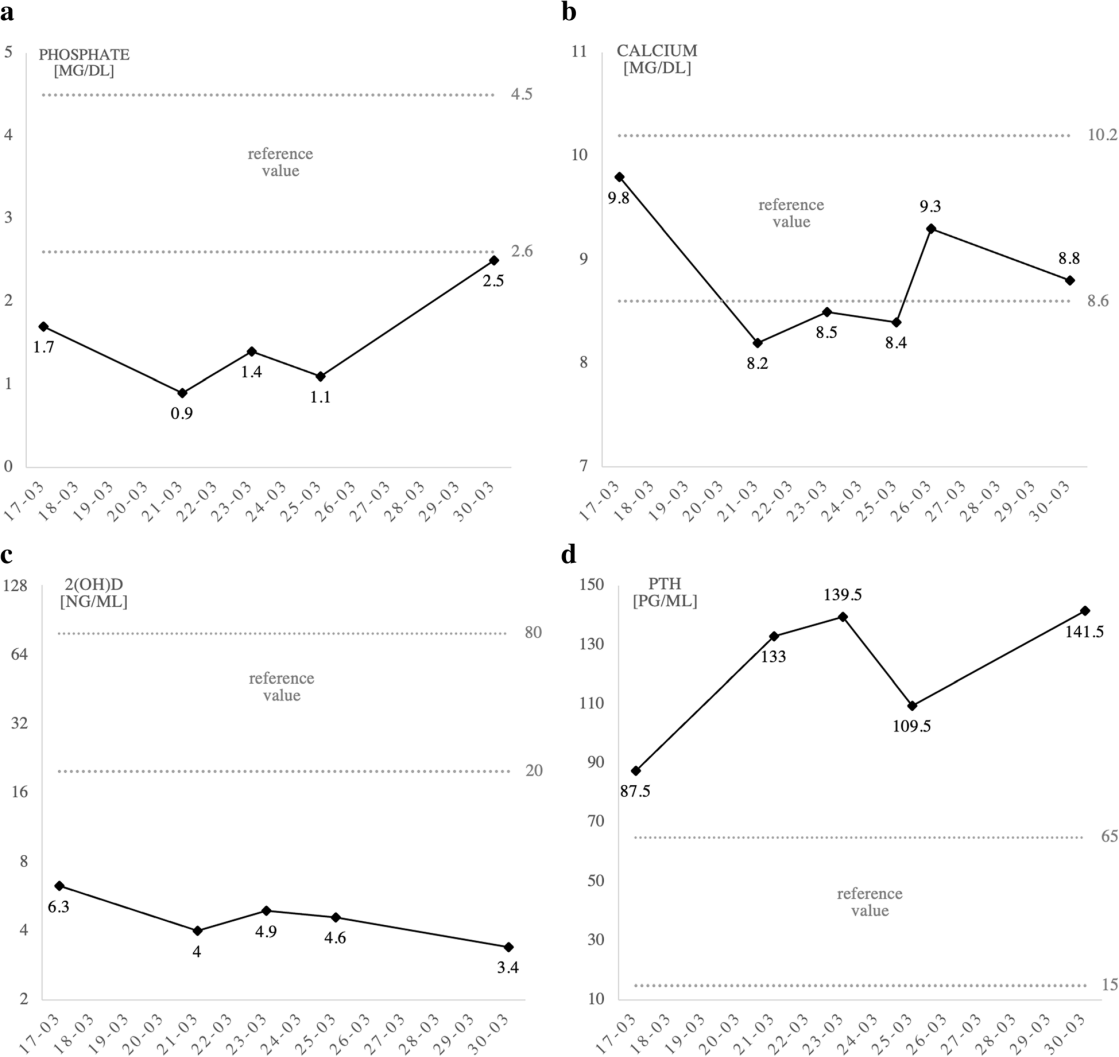
Perioperative biochemical response to the surgery performed on 20th March

**Fig. 5:**
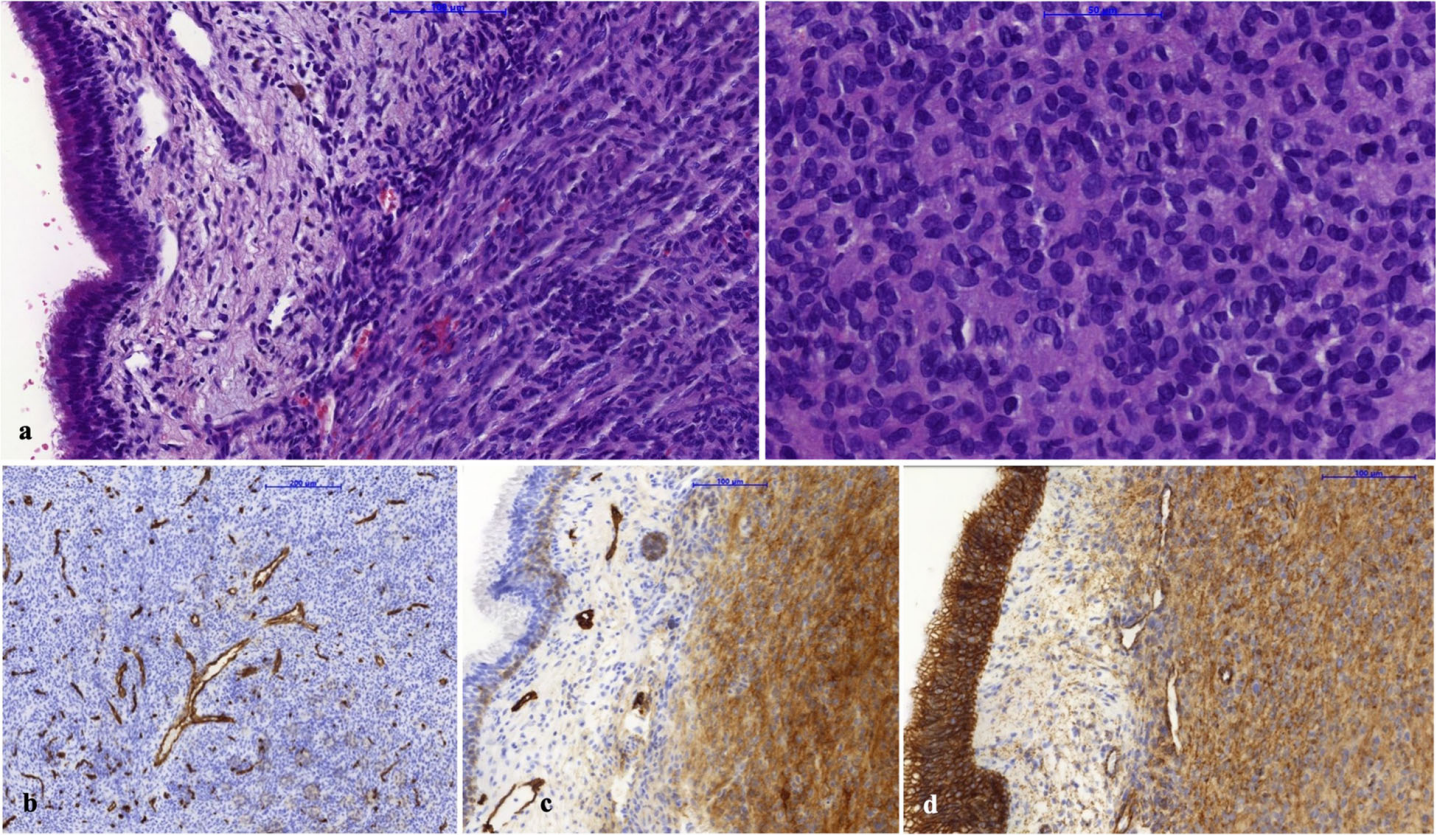
Hematoxylin and eosin staining revealed glomangiopericytoma infiltrating the paranasal sinus mucosa. The tumor consisted of oval and spindle cells forming solid sheets and whorls (**a**). CD34 immunohistochemical staining for CD34 showed no expression and highlighted tumor branching vasculature. Stains for CKAE1/3, EMA, S-100, desmin, SMA, HMB45 were also negative (**b**). Immunohistochemical reaction positivity with podoplanin (**c**) and cyclin-D (**d**)

**Fig. 6:**
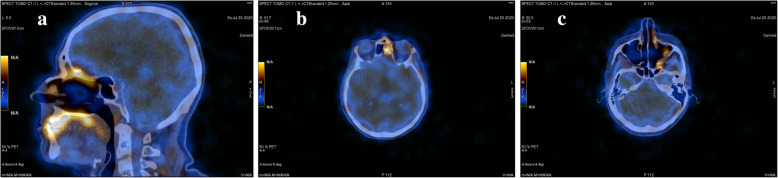
Post-surgery SPECT-CT somatostatin receptor scintigraphy scans showing slightly increased radiotracer uptake in the area of the left ethmoid region

Due to her symptoms, the patient repeatedly contacted health services and was hospitalized, including rheumatology, neurology and ENT departments, without establishing a diagnosis. Hypophosphatemia (1.98 mg/dl; normal ranges 2.5-4.5 mg/dl), vitamin D deficiency (7 ng/ml, normal range: 30-80 ng/ml), elevated levels of PTH (121.30 pg/ml, normal range: 18.40-80.10 pg/ml) and normocalcemia (total calcium 8.5 mg/dl, albumin-adjusted total calcium 9.3 mg/dl, normal range: 8.7-10.4 mg/dl; albumin 3.02 g/dl, normal range: 3.2-4.8 g/dl) were present in 2017 and 2018 in the patient’s hospital discharge summary reports. In April 2019 a biopsy of a tumor of the left nasal cavity was performed. The tumor had been incidentally found in a computed tomography (CT) scan and was histologically classified as “sinonasal hemangiopericytoma-like tumor”. Still, it had not been recognized as a possible cause of her systemic symptoms until November 2019 when she was referred to our Otorhinolaryngology Unit and a suspicion of oncogenic osteomalacia was raised. Serum FGF-23 level was elevated at 872 kRU/l (the upper limit of normal range: 110 kRU/l). CT showed a soft-tissue lesion opacifying the left ethmoid and frontal sinus, thinning medial wall of the left orbit and suggested possible intracranial tumor spread (Fig. 2A). Magnetic resonance imaging (MRI) confirmed the extension of the mass into the anterior cranial fossa (Fig. 2B). On admission to the Department of Otolaryngology for the surgical removal of the mass, her laboratory data were as follows: phosphate 1.7 mg/dl (2.6-4.5), calcium 9.8 mg/dl (8.6-10.2), 2(OH)D 6.3 ng/ml (20-80), PTH 87.5 pg/ml (15-65), ALP 279 U/l (35-104), creatinine 0.4 mg/dl (0.5-0.9) and a normal value of eGFR (>90 ml/min/1.73 m^2^). The total endoscopic endonasal resection of the tumor was performed in cooperation with a neurosurgeon with the use of a navigation system and a skull base defect was closed with the anterolateral thigh flap (Fig. 3). Lumbar drainage was placed after the surgery to lower the risk of cerebrospinal fluid leakage and removed on postoperative day 10. Due to anemia the patient received one unit of red cell concentrate. Cerebrospinal fluid leakage did not develop. The patient’s severe bone pain resolved rapidly after the surgery, but she did not notice any motor improvement at the time of hospital discharge. The fluctuations of laboratory parameters were observed (Fig. 4) and, unexpectedly, the level of FGF-23 measured on the 10th postoperative day was elevated at 1193 kRU/l. Histologically, the tumor was classified as glomangiopericytoma (Fig. 5). About a month after the operation her motor functions improved and soon she was able to walk with crutches outside and move without walking aids around the house. Due to high postoperative FGF-23 values and increasing serum PTH levels the patient was admitted to the Department of Endocrinology for further diagnosis. At that time biochemical evaluation revealed hypophosphatemia (2.4 mg/dl; 2.6-4.5), normocalcemia (9.6 mg/dl; 8.6-10.2), normal albumin (4.3 g/dl; 3.9-4.9) and total protein (7.4 g/dl; 6.4-8.3) levels, elevated alkaline phosphatase (301 U/l; 35-104) and PTH (149.8 pg/ml; 15-65) levels; serum 1,25 dihydroxyvitamin D concentrations rose to 16.8 ng/ml (20-80). 24-hour urine collection was performed for the assessment of phosphate (0.7 mg/24 h, normal range: 0.4-1.3/24 h) and calcium (82 mg/24 h, normal range: 100-250 mg/24 h) excretion. There were no signs of renal dysfunction (creatinine 0.4 mg/dl, normal range: 0.5-0.9 mg/dl; eGFR 104 ml/min, normal value: > 90 ml/min/1.73 m^2^; creatinine in 24-hour urine collection 1.1 g/24 h, normal range: 0.8-1.6 g/24 h; cystatin C 0.72 mg/l, normal range: 0.53-0.95 mg/l). SPECT-CT somatostatin receptor scintigraphy (SRS) with 99mTc-Tektrotyd for imaging showed moderate radiotracer uptake (the Krenning score of 1) in the left ethmoid region and could not exclude lesions extending to the left frontal sinus and frontal lobe intracranially (Fig. 6). The MRI result also indicated an incomplete tumor resection. The expression of somatostatin receptors was insufficient for the successful treatment of persistent disease with octreotide therapy.

**Fig. 7:**
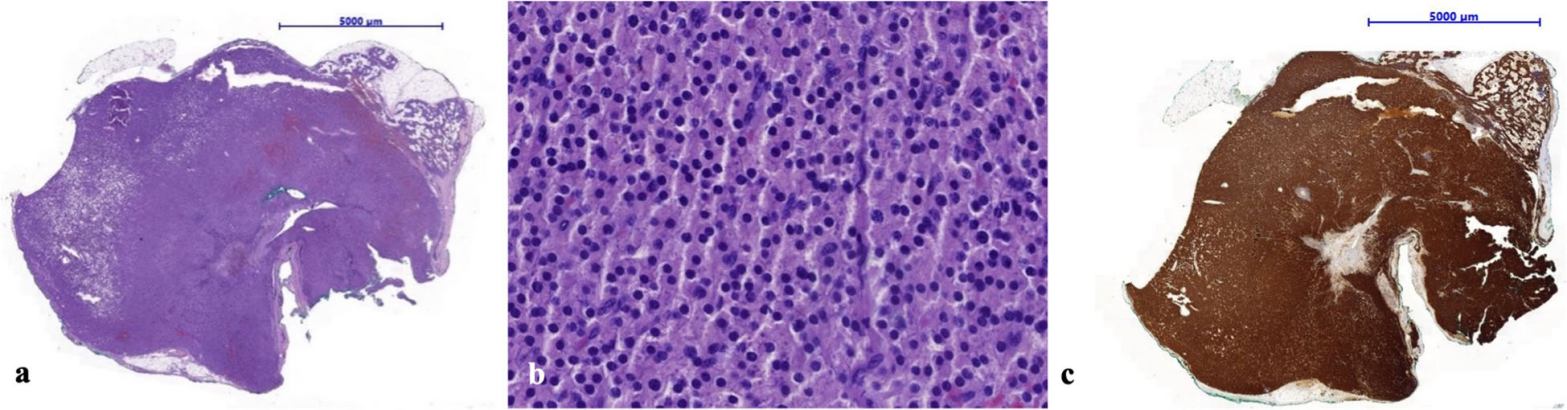
Histological sections of hematoxylin and eosin staining showing parathyroid adenoma characterized by a solid growth pattern, surrounded by a thin capsule, well demarcated from other tissues. At the periphery a rim of compressed normal parathyroid tissue admixed with fat cells is seen (**a**, **b**). Immunohistochemically, parathyroid adenoma stains intensely positive for chromogranin A (**c**)

Thyroid ultrasonography revealed a focal lesion behind the left thyroid lobe suggestive of an enlarged parathyroid gland. The cytopathologic evaluation of fine needle aspirate indicated a parathyroid tumor. The patient underwent the surgical resection of the pathological mass. The parathyroid glands were inspected intraoperatively and the complete excision of entire hyperfunctioning parathyroid tissue was confirmed using the MIAMI protocol (>50% drop of PTH measurement 10 min after the resection). A brown solid lesion of about 1.8 × 1.5 × 0.8 cm in size was histologically consistent with adenoma (Fig. 7). The record of the patient’s PTH showed that PTH value on the first day after the surgery (90.3 pg/ml; 15-65) was the lowest when compared to data collected over the past 6 months.

**Fig. 8:**
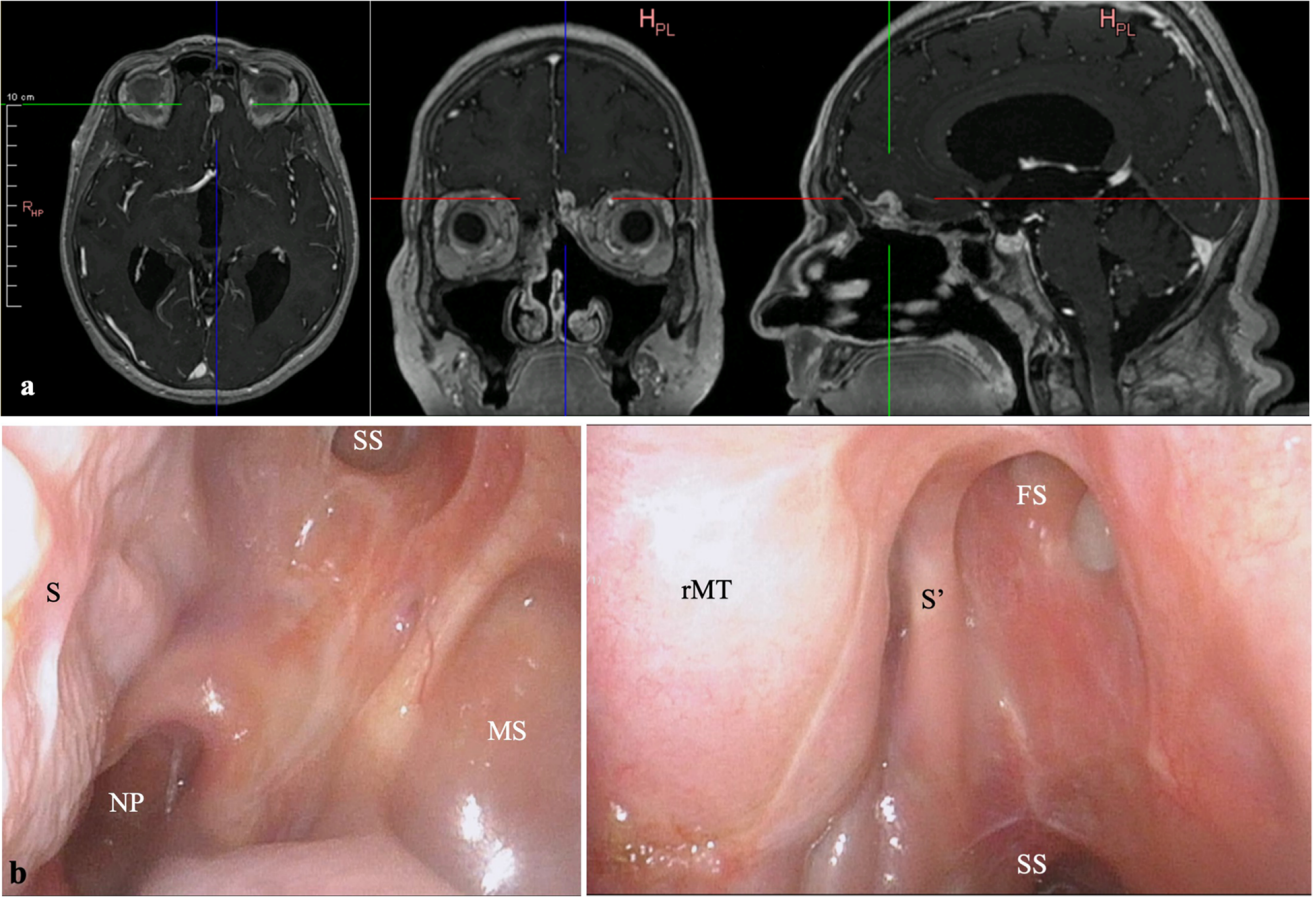
Follow-up 16 months postoperatively: MRI revealed an enhancing mass at the left anterior frontal skull base and the ethmoid roof, displaying a heterogeneous signal on T1- and T2-weighted sequence suspected of residual tumor, which did not correlate with the clinical and laboratory outcomes of the patient (**a**). Endoscopic view of the operation field with no sign of recurrence. Nasal septum (S), nasopharynx (NP), sphenoid sinus (SS), maxillary sinus (MS), right middle turbinate (rMT), partially resected nasal septum (S’), frontal sinus ostium with a small-sized polyp (FS) (**b**, **c**)

The patient walked without aids at follow-up 16 months after the surgery. Interestingly, laboratory evaluation revealed the normalization of FGF-23 (53 kRU/l). Due to the good clinical condition of the patient, the pharmacological or surgical treatment of residual sinonasal hemangiopericytoma has not been attempted. The woman is examined regularly at the Department of Otolaryngology and the Department of Endocrinology (Fig. 8).

## Discussion

Osteomalacia is characterized by deficient bone matrix mineralization and may be caused by the excess amount of FGF-23 produced by a tumor. Physiologically, FGF-23 is mainly expressed in osteoblasts and osteocytes [11], but it may be produced by neoplasms of mesenchymal origin (most commonly phosphaturic mesenchymal tumor mixed connective tissue variant [PMTMCT] or other rare entities including sinonasal hemangiopericytoma, osteosarcoma, hemangioma of bone, etc.) [12]. The tumors are small, slowly growing, and may be located within any soft tissue and bone area. Along with the non-specific symptoms of a paraneoplastic syndrome it is a cause of delayed diagnosis, which reached 20 years in extreme cases [12]. The majority of TIO-associated neoplasms are located in the lower extremities, followed by the head and neck region [3, 13], with sinuses being the predominant site [6, 14]. The surgical excision of the tumor with a wide margin is considered as the gold standard treatment [15]. If the tumor may not be detected or resected, the pharmacological treatment of TIO includes phosphate supplements and calcitriol or alphacalcidol [15].

Glomangiopericytoma, also called sinonasal hemangiopericytoma-like tumor, is a rare, borderline and low malignant potential neoplasm showing perivascular myoid phenotype, which comprises less than 0.5% of all nasal cavity and paranasal sinus tumors [16]. The cases of glomangiopericytoma inducing TIO were rarely reported in the literature [7]. The majority of glomangiopericytoma cases present clinically with unilateral nasal obstruction and/or recurrent epistaxis. Headache and vision impairment were rarely reported [17, 16]. Macroscopically, a soft, edematous, pinkish or reddish polypoid tumor may be easily mistaken for an inflammatory polyp [7]. The therapy of choice involves a radical surgical resection, preceded by preoperative embolization in some cases [17]. Persistent or recurrent disease resulting from incomplete tumor removal develops more frequently in intracranial and oral cavity lesions where *en bloc* tumor removal is challenging and is associated with a higher risk of life-threatening complications [6]. Due to the high recurrence rate reaching even 50%, regular post-operative follow-up is necessary including laboratory testing and diagnostic imaging [17]. According to literature data, the half-life of serum FGF-23 is short (8.5-58 min. depending on the study [18, 19]) and its levels decrease rapidly after the removal of the responsible tumor [13, 20, 21]. As regards our patient, postoperative FGF-23 serum concentrations were high above the reference range which (in correlation with imaging tests) suggested an incomplete hemangiopericytoma resection. However, FGF-23 levels eventually normalized, which may support the hypothesis that the percentage decline of FGF-23 levels is of greater importance than its absolute value in the early post-operative period [14]. It may also be due to individual variations in FGF-23 clearance[19].

**Fig. 9:**
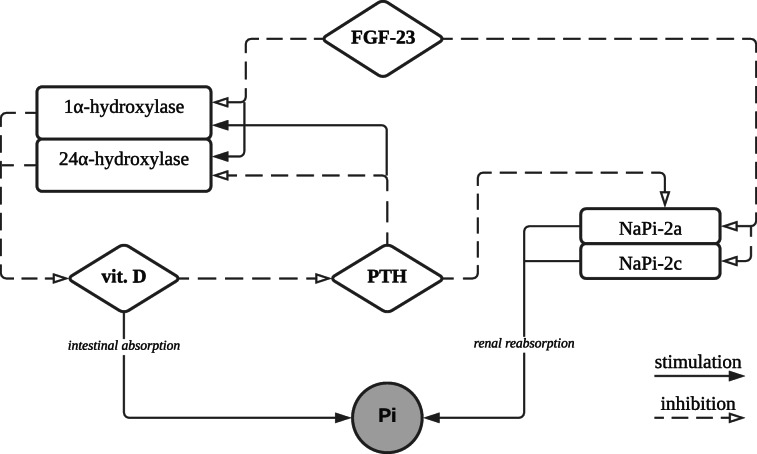
Regulation of phosphate homeostasis by FGF-23, vitamin D and PTH

In TIO, FGF-23 overproduction leads to urinary phosphate wasting and altered vitamin D metabolism. Phosphate is an abundant mineral in the body and has essential biological importance for energy metabolism, nucleic acid synthesis, bone mineralization and intracellular signaling [15, 22–25]. Phosphate homeostasis is maintained by a very complex interplay of endocrine factors including FGF-23, vitamin D and PTH[25] – the analysis of the tight hormonal interactions enables the understanding of the dramatic consequences of high circulating levels of FGF-23 (Fig. 9). FGF-23 directly causes phosphate loss by the reduction in type II sodium-phosphate cotransporters (NaPi-2a and NaPi-2c) in the renal proximal tubules [26]. NaPi-2a plays a major role in renal phosphate reabsorption (70-80%) and, thus, in the regulation of phosphate systemic balance [15, 27, 28]. Another essential action of FGF-23 is influencing the renal expression of the key enzymes of vitamin D metabolism, which leads to a significant decrease in 1,25(OH)_2_D_3_ serum level [11, 27]. This effect is mediated through the downregulation of 1α-hydroxylase, an enzyme that converts 25(OH)D_3_ to the hormonally active 1,25(OH)_2_D_3_, and the up-regulation of 24α-hydroxylase, catalyzing the degradation of both 25(OH)D_3_ and 1,25(OH)_2_D_3_ to inactive forms [25, 29, 30]. Since vitamin D is involved in approximately 30% of dietary phosphate absorption in the small intestine, its deficiency further contributes to negative phosphate balance [25, 31]. The role of vitamin D as the main regulator of calcium absorption in the gut is also of high importance [11, 32]. Inorganic calcium and phosphate are converted to hydroxyapatite crystals which mineralize bone matrix and give the skeleton its strength [11]. The deficiency of these elements, resulting from the above-mentioned processes, leads to osteomalacia.

This article presents a rather typical clinical course of the rare disorder, but with one very unusual feature – primary hyperparathyroidism.

PTH not only plays a central role in the maintenance of serum calcium levels, but also in phosphate regulation. Continuous exposure to PTH stimulates osteoclastic bone resorption in which both calcium and phosphate are mobilized from the bone [15, 33]. PTH inhibits renal phosphate reabsorption through the endocytosis of NaPi-2a [15, 27, 31], which is consistent with FGF-23 activity. However, unlike FGF-23, PTH increases the synthesis of 1,25(OH)_2_D_3_ by the stimulation of 1α-hydroxylase and the suppression of 24α-hydroxylase [24, 29]. Vitamin D exerts negative feedback on PTH production by suppressing its expression in the parathyroid gland [34].

Calcium and PTH levels in TIO are usually normal [1–6]. However, discrepancy in laboratory findings was observed [1, 7, 8]. In case of TIO and concomitant hyperparathyroidism, which occurred in the described case, the phosphate-lowering effect of FGF-23 was enhanced by PTH action causing high bone turnover and progressive bone loss. Interestingly, the appearance of patients with oncogenic osteomalacia resembles those with advanced hyperparathyroidism [15]. Hyperparathyroidism could be attributed to vitamin D deficiency which leads to hypocalcemia [9, 15, 29, 35–37]. An increase in PTH secretion is aimed to normalize 1,25(OH)_2_D_3_ serum concentrations (hypovitaminosis D diminishes negative feedback regulation on PTH) to maintain intestinal calcium absorption. The direct stimulation of parathyroid cells by a lower plasma Ca^2+^ level may be the second mechanism leading to increased PTH concentration [36]. During the years of the disease, our patient had few measurements recorded, in which calcium levels were slightly below the normal reference values. Cases of TIO and tertiary hyperparathyroidism were reported [38, 39] but only a few studies described the presence of parathyroid adenoma [37, 40–42]. Tertiary hyperparathyroidism is attributed to the prolonged high-dose phosphate treatment with the pathogenesis remaining unclear [9, 36, 38, 39]. Our patient did not receive phosphate supplementation. The process of polyclonal hyperplasia and the autonomization of parathyroid cells in response to chronic stimulation is known in the literature [37], as well as the possibility of their evolution into monoclonal adenoma [43–45]. It typically occurs in patients with chronic renal failure [46], a model resembling TIO. However, the pathogenesis of adenomatous transformation in patients with oncogenic osteomalacia needs to be elucidated [36]. It should be noted that after the excision of glomangiopericytoma in our patient, initially elevated PTH levels began to increase successively despite the stabilization of Ca^2+^ concentration. It might be the evidence of the suppressive effect of FGF-23 on PTH secretion [26, 47].

## Conclusions

TIO is an uncommon and acquired paraneoplastic condition developing due to the production of phosphaturic factor by the neoplasm, leading to impaired phosphate and vitamin D metabolism. The uniqueness of the presented case is the very rare coexistence of tumor-induced osteomalacia and parathyroid adenoma, most likely being a consequence of tertiary hyperparathyroidism. Further investigations of FGF-23 and PTH interplay should be conducted to elucidate the pathogenesis of hyperparathyroidism and tumorigenesis in some cases of TIO. By presenting this case, we wanted to highlight the importance of monitoring PTH concentrations in patients with TIO, as there is a possibility of masked hyperparathyroidism.

## Data Availability

The authors confirm that the data supporting the findings of this study are available within the article and its supplementary materials.

## Abbreviations

TIO: tumor-induced osteomalacia
FGF: fibroblast growth factor
PTH: parathyroid hormone
OOM: oncogenic osteomalacia
CT: computed tomography
SPECT: single-photon emission computed tomography
MRI: magnetic resonance imaging
RU: reference units
PMTMCT: phosphaturic mesenchymal tumor mixed connective tissue variant
